# Progression of macular retinoschisis following intravitreal aflibercept injection for myopic macular neovascularization—a case report and review of literature

**DOI:** 10.1186/s12886-024-03497-4

**Published:** 2024-05-28

**Authors:** Nikhil Gopalakrishnan, Aishwarya Joshi, Naresh Kumar Yadav, Vishma Prabhu, Snehal Bavaskar, Jay Chhablani, Ramesh Venkatesh

**Affiliations:** 1 Dept. of Retina and Vitreous, Narayana Nethralaya, #121/C, 1st R Block, Chord Road, Rajaji Nagar, Bengaluru, 560010 India; 2 University of Pittsburgh School of Medicine, Medical Retina and Vitreoretinal Surgery, 203 Lothrop Street, Suite 800, Pittsburg, PA 15213 USA

**Keywords:** Macular retinoschisis, Macular neovascularisation, Pathological myopia, Intravitreal anti-VEGF, Progression, Outcome

## Abstract

**Background:**

Macular retinoschisis (MRS) and myopic macular neovascularization (mMNV) are both potentially blinding complications of high myopia. In this case report, we highlight the progression of MRS after intravitreal anti-vascular endothelial growth factor (anti-VEGF) treatment for mMNV, as well as an extensive review of the literature on this topic.

**Case description:**

A 49-year-old woman presented with two weeks of recent onset blurring and metamorphopsia in her right eye. She had high myopia in both eyes (right eye − 20/60 with − 16D, left eye − 20/20 with − 13D). Slit-lamp ophthalmoscopy found a normal anterior segment in both eyes. On fundus examination, features of pathological myopia with posterior staphyloma and peripapillary atrophy were observed in both eyes. An active mMNV, as well as intraretinal fluid, minimal perifoveal inner and outer MRS, and focal posterior vitreous traction along the inferotemporal retinal arcade, were detected on optical coherence tomography (OCT) of the right eye. The patient received an intravitreal injection of Aflibercept (2 mg/0.05 ml).

**Results:**

OCT scans at two- and four-month follow-up visits revealed regressed mMNV with a taut epiretinal membrane, progressive worsening of outer MRS, and the development of multiple perifoveal retinal detachment inferior to the fovea. Pars plana vitrectomy surgery was performed for the progressive MRS with good anatomical (resolved MRS) and functional outcome (maintained visual acuity at 20/60) at the last one-month post-surgery visit.

**Conclusion:**

Intravitreal anti-VEGF injections for mMNV can cause vitreoretinal interface changes, exacerbating MRS and causing visual deterioration. Vitrectomy for MRS could be one of several treatment options.

## Introduction

Pathologic myopia lesions are characterized by retinoschisis, macular hole, epiretinal membrane (ERM), and myopic macular neovascularisation (mMNV) [1]. In eyes with pathologic myopia, mMNV is a vision-threatening complication [2]. Anti-vascular endothelial growth factor (anti-VEGF) intravitreal injection has been shown to effectively improve vision in patients with mMNV [3]. Myopic macular retinoschisis (MRS) is defined as the splitting of the retina in inner and/or outer layers with hyporeflective cystoid spaces and columnar structures, and it affects 9-34% of pathologic myopic eyes with posterior staphyloma [4, 5]. It is not uncommon for MRS to coexist with mMNV. In this report, we describe a case of progression of MRS following intravitreal anti-VEGF injection in an eye with pathological myopia and mMNV. While there are a few reports in the literature on this topic [6–9], the current report provides an overall understanding of the different pathogenetic mechanisms and risk factors for the progression and resolution of MRS in these high-myopia eyes with a typical case and a comprehensive review of the literature.

### Case description

**Fig. 1:**
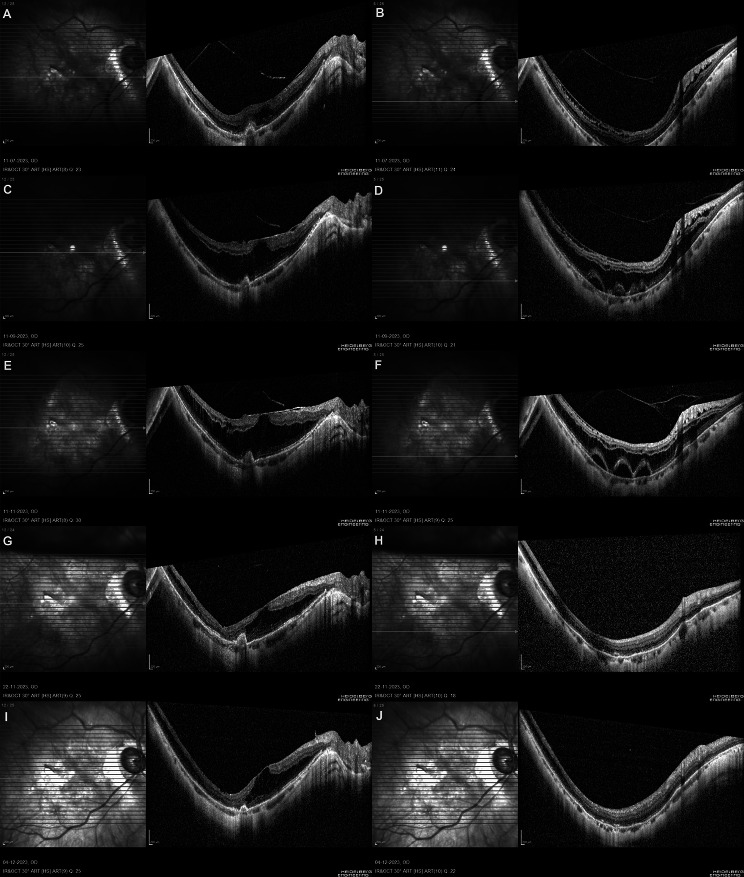
Horizontal volumetric macular optical coherence tomography (OCT) scans of the right eye in a patient with pathological myopia and myopic macular neovascularisation (mMNV) passing through the fovea and inferior retinal vascular arcade at different time points: **A, B**: OCT scan of the right eye at presentation reveals an irregular fusiform subretinal lesion with ill-defined margins, indicating mMNV, as well as intraretinal fluid, non-foveal involving minimal inner and outer macular retinoschisis (MRS), and focal posterior vitreous traction along the inferotemporal retinal vessel. **C, D**: Two months post intravitreal anti-VEGF therapy, OCT scans reveals the regressed mMNV with well-defined margins, epiretinal membrane and worsening of outer MRS and the development of multiple perifoveal retinal detachment inferior to the fovea. **E, F**: Four months post intravitreal anti-VEGF injection, OCT scans reveals further progression and worsening of MRS, as well as an increase in the size of the neurosensory detachment pockets. **G, H**: At 10 days post vitrectomy surgery, OCT scans reveal reduction in MRS and no reactivation of mMNV. **I, J**: At 30 days post vitrectomy surgery, similar OCT findings as noted in the last visit with no further deterioration of MRS

A 49-year-old woman presented to the retina clinic with recent onset visual complaints of blurring and metamorphopsia in her right eye that had been present for the past two weeks. Her right eye and left eye presenting visual acuities was 20/60 with − 16D and 20/20 with − 13D, respectively. Axial length measured was 28.16 and 27.35 mm in the right eye and left eye, respectively. Slit-lamp ophthalmoscopy found a normal anterior segment in both eyes, with clear lenses and normal intraocular pressure. Pathological myopia with posterior staphyloma and peripapillary atrophy in both eyes, with focal chorioretinal atrophy in the right eye, was noticed during a posterior segment examination of both eyes. Horizontal macular volume scans were obtained using the Spectralis optical coherence tomography (OCT) device (Heidelberg Engineering, Germany). OCT scan of the right eye revealed an irregular fusiform subretinal lesion with ill-defined margins, indicating mMNV, as well as intraretinal fluid, minimal perifoveal inner and outer MRS, detached posterior hyaloid at the fovea with a focal posterior vitreous traction along the inferotemporal retinal arcade (Fig. 1A, B). The patient was given an intravitreal Aflibercept (2 mg/0.05 ml) injection for the active mMNV and was asked to return in two months. At 2 months follow-up visit, the patient complained of persistent visual complaints and 20/60 vision in her right eye. The results of the fundus examination revealed no significant differences from the previous visit. At this visit, OCT scans revealed the mMNV with well-defined margins, indicating regression with worsening outer MRS and the development of multiple perifoveal retinal detachment inferior to the fovea. There was also an ERM over the macular region (Fig. 1C, D). Unable to explain the likely cause of the MRS’s progression and with no further deterioration in visual acuity, the patient was advised to observe with close self-monitoring of her visual symptoms and a fixed follow-up after 2 months. Two months later, her visual complaints had not improved, and her visual acuity remained at 20/60. At this visit, OCT scans revealed further progression and worsening of MRS, as well as an increase in the size of the neurosensory detachment pockets (Fig. 1E, F). Fluorescein angiography was used to detect activity from the existing mMNV, which revealed only staining and no active leakage from it.

Based on the visual symptoms, worsening MRS, presence of ERM and lack of reactivation of the existing mMNV, a 25-gauge pars plana vitrectomy surgery was performed, which included complete removal of the posterior cortical vitreous, Brilliant Blue G-assisted peeling of ERM along with complete removal of the internal limiting membrane at the macula and intraocular gas endotamponade with 20% sulfur hexafluoride. Follow-up visits at 10 days and 30 days after surgery revealed resolution of patient symptoms, maintenance of visual acuity at 20/60, and reduction in MRS and regressed pre-existing mMNV on OCT (Fig. 1G-J). The patient provided informed consent for the sharing of clinical information and the use of images for publication and research purposes.

## Discussion

According to studies, MRS can be stable for years or may naturally progress to develop a full-thickness macular hole or foveal retinal detachment [10–12]. These complications arise from changes at the vitreoretinal interface. Similarly, there are enough reports in the medical literature to show progression in MRS after treatment with intravitreal anti-VEGF injections for mMNV. Shimada et al. reported that 5.4% of 74 eyes treated with intravitreal bevacizumab injection for mMNV developed macular retinal detachment after treatment, and four eyes developed foveoschisis around the mMNV [13]. Huang et al. detected MRS progression in seven of the 11 patients with preexisting ERM and MRS, but no progression in the six contralateral eyes with MRS and ERM but not treated with intravitreal anti-VEGF [8]. According to Lai et al., two of the 37 eyes had MRS progression and one eye had full thickness macular hole after intravitreal bevacizumab or ranibizumab injections [9]. Ceklic et al. found new MRS in eight of 474 eyes (1.7%) in the outer retinal layers, and new MRS developed in seven intravitreal ranibizumab-treated eyes but only one untreated fellow eye. During the 12-month follow-up period, none of the eight eyes had MRS progress to a full-thickness macular hole or foveal detachment [6]. In 122 mMNV eyes treated with Conbercept, Zhou et al. reported ten eyes with MRS progression and two eyes with new-onset MRS [7]. Tsui et al. identified risk and protective factors for the progression and development of MRS in eyes treated with intravitreal anti-VEGF injections for mMNV [14]. 

Anti-VEGF injections can cause liquefaction of vitreous and induce posterior vitreous detachment, stimulate ERM proliferation, influence vitreous adhesion or traction on the retina, potentially change the density of the vitreous humor and cause shrinkage of the fibrovascular tissue, and may result in tractional forces and enhance the separation of different retinal layers, all of which can lead to the occurrence or progression of MRS. Tsui et al. also identified risk factors for the progression and development of MRS, such as the presence of outer retinal layer schisis, ERM, lamellar macular hole, and male sex at baseline [14]. The presence of outer retinal layer schisis at the baseline, as well as the development of a new ERM, tangential forces exerted by the ERM, and contraction of the fibrovascular tissue following anti-VEGF injection, may be responsible for the progression of MRS and the development of focal retinal detachment in this case. The study conducted by Tsui et al. revealed that the administration of intravitreal ranibizumab injection demonstrated a protective effect against vision-threatening MRS [14]. The authors proposed that the varying fibrotic effects observed with different anti-VEGF injections may be associated with varying probabilities of developing vision-threatening MRS. Ranibizumab might exhibit a potentially reduced fibrotic effect when compared to other potent anti-VEGF agents, suggesting its potential protective role against the progression of MRS. In the present study, a single intravitreal injection of Aflibercept was administered, which is recognized as a potent anti-VEGF agent due to its capacity to induce early fibrosis of the MNV.

MRS can be resolved through a variety of mechanisms, including complete posterior vitreous detachment and rupture of the internal limiting membrane. These processes can occur naturally during the course of the disease or be induced by intravitreal injections or performing vitrectomy surgery [13–15]. Tsui et al. found a higher rate of MRS resolution after intravitreal anti-VEGF agent therapy compared to the 3.9% reported in the natural course of MRS study, indicating that intravitreal anti-VEGF plays an important role in changing the vitreoretinal interface and MRS resolution [13, 14]. For more than a decade, surgical management of MRS with or without associated foveal detachment, full-thickness macular hole, or even macular hole-related retinal detachment with pars plana vitrectomy, posterior cortical vitreous separation, and internal limiting membrane peeling has resulted in favourable anatomical and visual outcomes [16–18]. In this case, we waited four months for the progressive MRS to resolve spontaneously. During this time, we could have considered intravitreal injections to induce posterior vitreous separation and achieve faster MRS resolution. However, after identifying a taut ERM and a regressed mMNV on follow-up OCT scans, a more aggressive decision was made to perform a pars plana vitrectomy to actively release the tractional forces on the vitreoretinal interface, resulting in a successful anatomical and functional outcome.

In conclusion, this case, along with a thorough review of the literature, emphasizes the role of vitreoretinal interface changes caused by intravitreal anti-VEGF therapy in MRS progression and discusses the various methods for MRS resolution.

## Data Availability

No datasets were generated or analysed during the current study.

## Abbreviations

mMNV: Myopic macular neovascularization
ERM: Epiretinal membrane
VEGF: Vascular endothelial growth factor
MRS: Macular retinoschisis
OCT: Optical coherence tomography
